# Bacterial Pathogens and Their Antimicrobial Resistance in Farmed Nile Tilapia Experiencing “Summer Mortality” in Kafr El-Sheikh, Egypt

**DOI:** 10.3390/microorganisms13112448

**Published:** 2025-10-25

**Authors:** Merna M. A. Hassan, Riad H. Khalil, Mahmoud M. Abotaleb, Mahmoud T. Amer, Hany M. R. Abdel-Latif

**Affiliations:** 1 Department of Poultry and Fish Diseases, Faculty of Veterinary Medicine, Alexandria University, Alexandria 22758, Egypt; merna.diab@alexu.edu.eg (M.M.A.H.); riad.khalil@alexu.edu.eg (R.H.K.); mahmod.amer@alexu.edu.eg (M.T.A.); 2 Central Laboratory for Evaluation of Veterinary Biologics, Agriculture Research Center (ARC), Cairo 11381, Egypt; m.abotaleb84@yahoo.com

**Keywords:** surveillance, outbreaks, pathogens, antibiotic resistance, Nile tilapia

## Abstract

During four outbreaks in 2023 and 2024, samples from pond-reared Nile tilapia were taken from different farms located in Kafr Elsheikh governorate, Egypt. Samples were submitted for laboratory examinations. Diseased fish exhibited bacterial septicemia and some cases died without showing any clinical signs. A total of 30 bacterial isolates were isolated and identified. Of these isolates, 57% were identified as Gram-positive bacteria, whereas the remaining 43% were identified as Gram-negative bacteria. PCR targeting the 16S rRNA gene and genome sequencing confirmed five bacterial isolates as *Aeromonas veronii* (30%), *Vibrio alginolyticus* (13.3%), *Enterococcus faecalis* (23.3%), *Aerococcus viridans* (16.7%)*,* and *Staphylococcus epidermidis* (16.7%). The NCBI GenBank accession numbers of these strains were (PV018985) for *A. veronii*, (PV016854) for *V. alginolyticus*, (PV013413) for *E. faecalis*, (PV032005) for *A. viridans*, and (PV012491) for *Staph. epidermidis*. The antibiogram revealed that the bacterial strains showed resistance to most of the antibiotics tested. *A. viridans* exhibited resistance to nearly all the antibiotics except for intermediate sensitivity to ciprofloxacin and amoxycillin/clavulanic acid. However, *A. veronii* showed high sensitivity to amoxycillin/clavulanic acid, oxytetracycline, kanamycin, and trimethoprim/sulfamethoxazole and intermediate susceptibility to ciprofloxacin. Similarly, *E. faecalis* showed high susceptibility to amoxycillin/clavulanic acid, ciprofloxacin, and trimethoprim/sulfamethoxazole in addition to intermediate sensitivity to ampicillin and kanamycin. Furthermore, *Staph. epidermidis* strain was highly susceptible to ampicillin, amoxycillin/clavulanic acid, oxytetracycline, novobiocin, and trimethoprim/sulfamethoxazole and was partially sensitive to kanamycin and ciprofloxacin. To conclude, summer mortalities recorded in farmed tilapia were closely related to a multifactorial bacterial origin with different sensitivity to antibiotic discs.

## 1. Introduction

Nile tilapia (*Oreochromis niloticus*) is one of the most farmed fish worldwide [1]. In Egypt, it is the cornerstone of fish farming and one of cheapest fish species [2,3]. Globally, Egypt is one of the top tilapia producers in Africa and ranks as the third largest producer of farmed tilapia after China and Indonesia, accounting for 17.4% of the world total production [4,5]. Kafr El-Sheikh province is a leading contributor to Egypt’s aquaculture industry, providing 324,479 tons, which accounts for 55% of overall production. It contributes with 259,583 tons in relation to the total production [6]. However, the expansion of the tilapia industry faces significant constraints due to the scarcity of freshwater resources and the frequent occurrence of disease outbreaks [7]. In Egypt, agricultural drainage water is permitted for use in aquaculture. This practice has serious consequences on the water quality, as it transfers various contaminants directly into the production systems [8]. The excessive use of agricultural fertilizers and pesticides is a dangerous problematic issue, which, in turn, pollutes surface and groundwater sources involving nitrates from fertilizers and bacteria from livestock waste [9,10]. It also increases the risk to fish health and plankton populations [11]. Regarding this issue, Ali et al. [12] declared a strong linkage between the poor water quality and the occurrence of tilapia mortalities in Egypt.

The increase in water temperatures during summer also has a great impact on flourishing bacterial fish pathogens directly by changing their biological properties or indirectly by changing the diversity of fish that are influenced. This may facilitate those bacteria to localize, proliferate, and enter fish tissues, which could lead to increasing the disease morbidity and mortality [13].

Egyptian fish farms have experienced multiple records of mass mortality outbreaks, mainly during the summer season, with substantial losses in farmed tilapia [14]. In this regard, Abdel-Moneam et al. [15] reported that the reason for the fish kills seemed to be attributed to multifactorial etiologies, which were triggered by various environmental factors. These authors declared that inferior water quality such as extreme levels of unionized ammonia and high water temperature have contributed to tilapia mortalities due to several bacterial pathogens such as *Aeromonas hydrophila*, *Vibrio alginolyticus*, and *Vibrio cholerae* in Kafr El-Sheikh. Several other researchers explained that “Summer Mortality” in tilapia is linked to a viral pathogen, as Fathi et al. [16] found that tilapia lake virus (TiLV) has been detected in tilapia affected by summer mortality in Egypt. On the other hand, Abbas et al. [17] described a case report of summer mortality syndrome in tilapia that was not linked to TiLV but caused mainly by pathogenic bacteria identified as *Streptococcus agalactiae*, *A. hydrophila*, and *V. cholera*. Similarly, Elsheshtawy et al. [18] proved that *A. hydrophila* was the causative agent of summer mortalities in semi-intensive tilapia farms in Kafr El-Sheikh. Nonetheless, disease eruptions in fish farms are not restricted to the summer months, but they could occur throughout the year. For instance, co-infections of *V. alginolyticus*, *Aeromonas* spp., and *Enterococcus faecalis* were recorded in early autumn during heavy mortalities of poly-cultured Nile tilapia and African catfish [19]. According to these authors, bad water quality represented by high concentrations of ammonia, nitrite, and nitrate together with high bacterial counts in water make cultured fish more vulnerable to bacterial infections. *A. hydrophila*, *Pseudomonas fluorescens*, and *Streptococcus iniae* were also isolated during spring, summer, and autumn seasons from 12 tilapia and carp farms experiencing severe mortalities in Kafr El-Sheikh [20]. Moreover, Enany et al. [21] identified *A. hydrophila*, *Ps. fluorescens*, and *V. cholera* strains in moribund tilapia during summer and winter mortalities.

Antibiotics were frequently prescribed for combating bacterial infections and even as prophylactics to reduce the occurrence of bacterial diseases of fish [22,23]. However, the inappropriate and extensive use of antibiotics directly contributes to the development of resistant bacteria in the aquatic environment [24]. This can also increase antibiotic resistance in fish pathogens and transfer this resistance also to bacterial pathogens affecting humans and land animals [25]. Antibiotic residues in aquatic products (fish fillets and their processed products) also pose high health risks to human beings [26]. Herein, the present study was carried out to investigate and characterize the pathogens, of bacterial origin, that are associated with tilapia mortality outbreaks in some Egyptian fish farms located in Kafr El-Sheikh province. Antibiotic sensitivity testing has also been carried out to effectively find the best chemotherapeutic for controlling the diagnosed bacterial infections, combating the problem of antibiotic resistance, and guiding effective fish health strategies.

## 2. Materials and Methods

### 2.1. Outbreak Reporting and Case Detection

Sudden outbreaks associated with high mortalities have been recorded in farmed Nile tilapia at different localities in Kafr El-Sheikh province, Egypt. A total of 3 outbreaks occurred during summer seasons (2023 and 2024) in different private farms with a history of recorded mortalities during the last few years. A single outbreak has been reported in late spring of 2024. The sampled fish were reared in earthen ponds supplied with agricultural drainage from the Kafr El-Sheikh, Egypt. Diseased fish were off food, lethargic, and exhibited signs of bacterial septicemia. Of these, moribund specimens of average body weight (80.0 ± 20 g) from each region were sampled and transported on ice immediately to the Microbiology Unit at Alexandria Provincial Lab, Animal Health Research Institute, Agriculture Research Center, Alexandria, Egypt, for further examinations. Clinical and postmortem examinations were performed according to guidelines outlined by Noga [27].

### 2.2. Bacteriological Examination

Liver, kidney, and spleen from each specimen were aseptically cultured onto Tryptic Soy Broth (TSB; Difco^TM^, Detroit, MI, USA) for pre-enrichment, incubated overnight at 30 °C. Sterile loopfuls were streaked onto Tryptic Soya Agar (TSA; Difco^TM^) supplemented with 5% sheep blood to examine the hemolytic activity of the retrieved bacterial isolates. Loopfuls were also cultured on Thiosulphate Citrate Bile Salts Sucrose agar (TCBS; HiMedia^TM^) (the selective media for Vibrio species), Nutrient Agar (NA; Difco^TM^), and Brain Heart Infusion Agar (BHIA; Difco^TM^). Cultured plates were incubated for 24–48 h at 28 °C. Pure colonies were phenotypically characterized by culture morphology, Gram stain, and cell motility in semisolid media [28]. Consequently, biochemical identification of the retrieved isolates was carried out using cytochrome oxidase test, catalase test, triple sugar iron (TSI) test, salt tolerance test (NaCl 4%, 6%, 8%, and 10%), and VITEK^®^ 2 Compact microbial identification system (BioMérieux, Marcy l’Etoile, France) following the manufacturer’s instructions. The purified isolates were cultured in TSB supplemented with 20% (*v*/*v*) glycerol and preserved at −80 °C for further molecular identification.

### 2.3. Molecular Screening of Bacterial Isolates

#### 2.3.1. Genomic DNA Extraction

A total of 5 different pure-cultured bacteria were randomly selected. Molecular examination of these isolates was performed at the Central Laboratory for Evaluation of Veterinary Biologics, Agriculture Research Center, Cairo, Egypt. Briefly, the bacterial isolates were streaked onto TSB and incubated at 28 °C for 48 h. The bacterial DNA was extracted with QIAamp DNA mini kit, Catalogue No. 51304, USA, according to the manufacturer’s protocol.

#### 2.3.2. PCR Using the Universal Bacterial Primer

According to the Supplementary Material (Table S1), PCR was performed using the universal 16S bacterial primers according to Lagacé et al. [29]. After amplification, 20 μL of PCR products were electrophoresed in 1.5% agarose gel, stained with ethidium bromide, photographed by gel documentation system, and the data was analyzed through computer software. Subsequently, amplicons were purified using QIA quick PCR Purification kit (QIAGEN, Germantown, MD, USA). *Aeromonas* spp. 16S rRNA gene (953 bp) was amplified using the DNA extracted from one bacterial isolate as reported by Gordon et al. [30]. The PCR products were electrophoresed, analyzed, and purified as mentioned earlier. Finally, a fraction of the purified PCR product was used for sequencing.

PCR amplification with *Vibrio*-specific primer (*Vibrio*-specific 16S rRNA) was used to amplify 663 bp from the general bacterial 16S rRNA as specified by Tarr et al. [31]. After PCR product analysis, the amplified region of the target gene was purified and prepared for sequencing.

### 2.4. Sequencing and Phylogenetic Analysis

The purified 16S rRNA gene fragments were sequenced using Applied Biosystems 3130 automated DNA Sequencer and a BigDye Terminator V3.1 cycle sequencing kit (Perkin-Elmer/Applied Biosystems, Foster City, CA, USA, Cat. No. 4336817). The sequencing procedures were performed at the National Laboratory for Veterinary Quality Control of Poultry Production in Giza, Egypt. The obtained sequential data were assembled, identified, and aligned against other available sequences published in the GenBank database using the BLAST search. The 16S rRNA gene (663, 953, and 1458 bp) sequences of the five selected bacterial isolates were deposited in GenBank. The phylogenetic analysis of consensus sequences was constructed by MEGA 12.0 software using the maximum-likelihood method and the confidence level was verified by bootstrap test for each branch at 1000 replicates [32].

### 2.5. Antibiotic Susceptibility Test

The antibiogram of the identified bacterial strains were examined using the disc diffusion methodology. The tested antimicrobial discs were penicillin (P, 10 µg), ampicillin (AMP, 10 µg), amoxycillin/clavulanic acid (AMC, 20/10 µg), trimethoprim/sulfamethoxazole (COT, 25 µg), ciprofloxacin (CIP, 5 µg), oxytetracycline (O, 30 µg), erythromycin (E, 15 µg), novobiocin (NV, 30 µg), and kanamycin (K, 30 µg). The antibiotic discs were placed on Mueller–Hinton agar (Oxoid^TM^, Detroit, MI, USA) plates and incubated for 24 h at 25 °C. Inhibition zone diameters were measured, and the results were categorized as either susceptible (S), intermediate (I), or resistant (R), following the criteria established by the Clinical and Laboratory Standards Institute [33].

## 3. Results

### 3.1. Clinical Signs and Necropsy Findings

**Figure 1:**
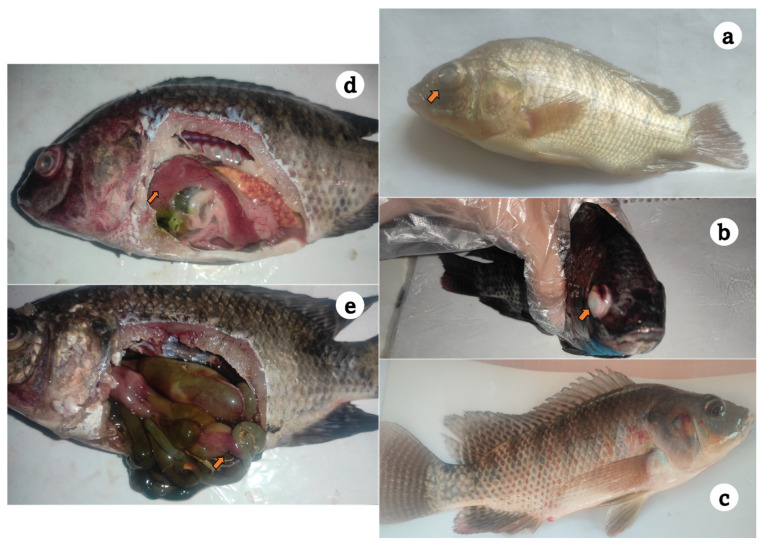
Diseased Nile tilapia sampled from private farms at Kafr Elsheikh governorate showing unilateral exophthalmia (**a**), darkened skin color and corneal opacity (**b**), surface hemorrhages over the fish skin and hemorrhagic vent (**c**), friable and congested liver (**d**), and enteritis, inflamed intestinal tract, and yellow abdominal dropsy (**e**).

The surviving fish had a history of anorexia, surface swimming, loss of equilibrium with no reflexes, and sudden onset of high mortalities. As shown in Figure 1, moribund and recently dead fish showed hemorrhagic patches over the skin, darkened skin color, unilateral exophthalmia, corneal opacity, eroded fins, and dropsy, and certain cases died without any signs. Internally, signs included congested kidney, enlarged friable liver, and enteritis with yellowish ascitic fluid that flowed out after dissection.

### 3.2. Distribution and Diversity of Bacterial Isolates

According to Table 1, a total of 30 (*n*) bacterial isolates were recovered from the examined tilapia specimens, which were phenotypically identified following the standard protocol described for bacterial isolation and identification. Out of the retrieved bacteria, 17 isolates were identified as Gram-positive bacteria, while the other 13 isolates were Gram-negative bacteria. The bacterial isolates were grouped into five different genera: *Aeromonas* spp., *Vibrio* spp., *Aerococcus* spp., *Enterococcus* spp., and *Staphylococcus* spp. The first outbreak was documented during the summer of 2023, in which the isolated bacteria was identified as *Aeromonas veronii* (*n* = 9). The second case occurred in spring of 2024, which was linked to *Aerococcus viridans* (*n* = 5). On the other hand, co-infecting pathogens have been reported in several fish samples of the third case, including *Enterococcus faecalis* (*n* = 7) and *Staphylococcus epidermidis* (*n* = 5) during the summer of 2024. In the last outbreak, *Vibrio alginolyticus* (*n* = 4) were retrieved from diseased fish during summer season of the same year. The distribution of these bacteria in various tissues of infected fish was (*n* = 13) in the liver, followed by spleen (*n* = 9) and kidney (*n* = 8).

*A. veronii*, *A. viridans*, and *E. faecalis* showed α-hemolytic colonies on blood agar, while *Staph. epidermidis* revealed β-hemolytic activity and *V. alginolyticus* showed no hemolysis. Characteristic swarming and large yellow colonies of *V. alginolyticus* were also detected on TCBS selective media. Subsequently, light microscopy examination of recovered isolates revealed Gram-negative, short bacilli (*A. veronii*) or comma-shaped rods (*V. alginolyticus*) as well as Gram-positive spherical to ovoid cocci arranged in pairs (*E. faecalis*), long chains (*A. viridans*), or forming grape-like clusters (*Staph. epidermidis*).

### 3.3. Biochemical Characteristics of Bacterial Isolates

As summarized in Table 2, *A. veronii* were identified as oxidase- and catalase-positive, and the TSI test showed K/A reaction (alkaline slant and acidic butt) with no gas production. Similarly, *V. alginolyticus* were oxidase- and catalase-positive, while the TSI test showed A/A reaction (acidic slant and acidic butt) without gas production. These findings confirm the identity of both *A. veronii* and *V. alginolyticus*, which are in accordance with the expected phenotypic characteristics. Moreover, both *E. faecalis* and *A. viridans* were recorded as oxidase- and catalase-negative, whereas *Staph. epidermidis* were oxidase-negative and catalase-positive. Of interest, all bacterial strains showed salt tolerance up to 8% NaCl.

Based on VITEK 2 biochemical identification, two isolates were confirmed and identified as *E. faecalis* with a probability estimated by 97% and *Staph. epidermidis* with a probability of 99% as reported in Supplementary Material (Table S2). The biochemical profile of *E. faecalis* showed typical positive reactions to production of Arginine dihydrolase2 (ADH2s), Pyrrolidonyl-arylamidase (PyrA), fermentation of ribose, sorbitol, trehalose, maltose, mannitol, mannose, and growth in media containing 6.5% NaCl. However, it tested negative for raffinose and lactose utilization, urea hydrolysis, and phosphatase production. The examined *Staph. epidermidis* was classified based upon positive reactions to urea hydrolysis, phosphatase production, utilization of lactose, mannose, and maltose, as well as growth in media containing 6.5% NaCl. Furthermore, this bacterial strain was typically negative to novobiocin resistance. It was also negative for ribose, sorbitol, trehalose, mannitol, and raffinose utilization tests. Based on the morphological and biochemical characters, five different bacterial strains were selected for additional molecular analysis.

### 3.4. 16S rRNA PCR, Sequencing, and Phylogenetic Analysis

PCR positively amplified the 1485 bp band of the 16S universal gene of *A. viridans*, *E. faecalis*, and *Staph. epidermidis*. Moreover, amplification bands of 953 bp and 663 bp of the 16S rRNA gene were detected specific to *A. veronii* and *V. alginolyticus*, respectively (Supplementary Material—Figures S1 and S2). The obtained sequences of these five isolates were deposited and published on GenBank database. The accession number of submitted nucleotide sequences were PV018985 for *A. veronii*, PV016854 for *V. alginolyticus*, PV013413 for *E. faecalis*, PV032005 for *A. viridans*, and PV012491 for *S. epidermidis*. BLAST analysis displayed high similarity and query coverage between our bacterial isolates and similar 16S rRNA sequences available on GenBank. To investigate evolutionary relationships, three maximum-likelihood phylogenetic trees were constructed based on 16S rRNA sequences using MEGA version 12.

**Figure 2:**
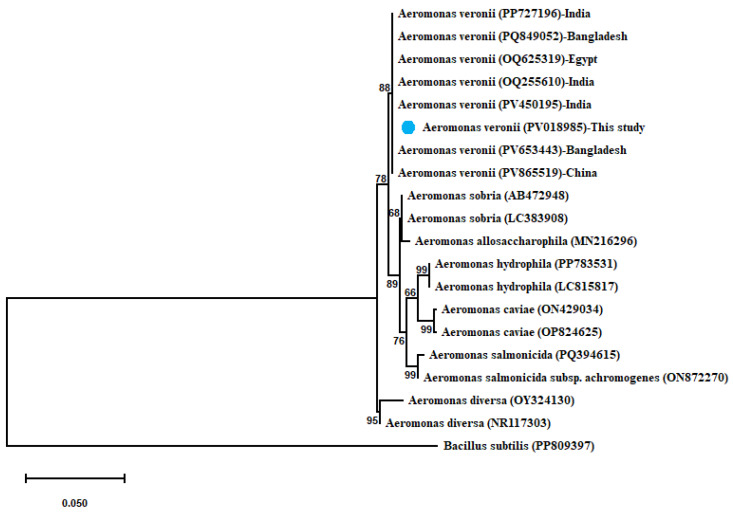
Phylogenetic tree showing clear clustering of the partial 16S rRNA gene sequence of *A. veronii* with other related partial 16S rRNA gene sequences of *Aeromonas* species with out-group Bacillus subtilis uploaded from the GenBank database. Phylogenetic analyses were conducted with MEGA12.0 software, using the maximum-likelihood method with Hasegawa–Kishino–Yano model with Gamma distribution (G). Percentage bootstrap values (1000 replicates) are shown at each branch point. The scale bar 0.050 represents substitutions per nucleotide position. The isolate recovered in the present study is marked with a solid blue circle.

*A. veronii* showed 100% similarity with (OQ625319) from Egypt, (PP727196–OQ255610–PV450195) from India, (PQ849052–PV653443) from Bangladesh, and (PV865519) from China. The derived maximum-likelihood phylogenetic tree showed clear clustering of the sequenced 16S rRNA gene of *A. veronii* with their related sequences and separated from other *Aeromonas* spp., as shown in Figure 2.

**Figure 3:**
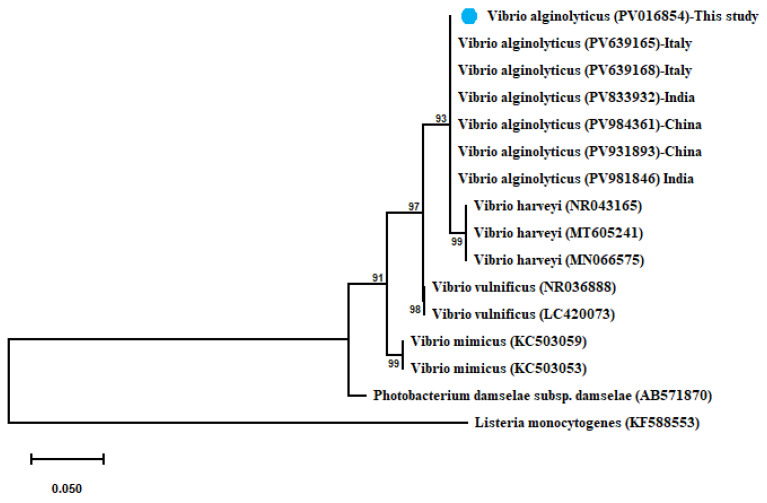
Phylogenetic tree showing the relationship of the partial 16S rRNA gene sequence of *V. alginolyticus* with other related partial 16S rRNA gene sequences of *Vibrio* species with *Listeria monocytogenes* as out-group uploaded from the GenBank. Phylogenetic analyses used the maximum-likelihood method with the Kimura 2-parameter model with Gamma distribution (G). Percentage bootstrap values (1000 replicates) are shown at each branch point. The scale bar 0.050 represents substitutions per nucleotide position. The isolate recovered in the present study is marked with a solid blue circle.

*V. alginolyticus* shared 100% identity with (PV639165–PV639165) from Italy, (PV981846–PV833932) from India, and (PV984361–PV931893) from China. A maximum-likelihood-based phylogenetic tree revealed the evolutionary relatedness of the sequenced *V. alginolyticus* and their related sequences combined with another *Vibrio* spp., as represented in Figure 3.

**Figure 4:**
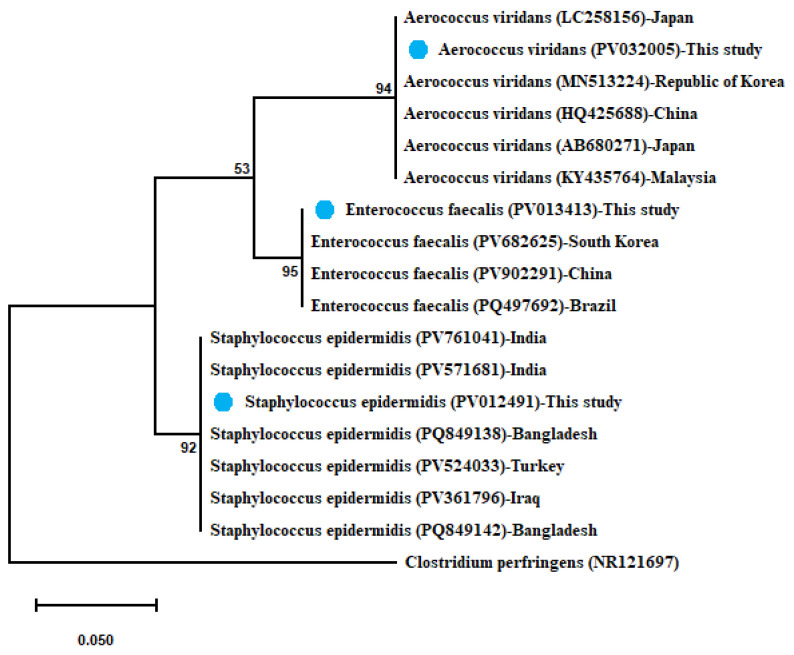
Phylogenetic tree showing clear clustering of 16S rRNA partial sequences of *E. faecalis*, *A. viridans*, and *Staph. epidermidis* with out-group *Clostridium perfringens* retrieved from GenBank database. The maximum-likelihood method used the Jukes–Cantor model with uniform rates. The support level in percentage, after 1000 repetitions, is indicated next to each branch. The scale bar 0.050 represents substitutions per nucleotide position. The isolates recovered in the present study are marked with solid blue circles.

In the same way, *A. viridans* showed 100% similarity with (HQ425688) from China, (AB680271) from Japan, (MN513224) from Republic of Korea, and (KY435764) from Malaysia, and this strain was 99.85% similar to (LC258156) from Japan. Moreover, *E. faecalis* shared 100% identity with (PQ497692) from Brazil and was 99.86% similar to (PV902291) from China and (PV682625) from South Korea. The sequenced *S. epidermidis* exhibited 100% similarity with (PV524033) from Turkey, (PV761041–PV571681) from India, (PQ849138–PQ849142) from Bangladesh, and (PV361796) from Iraq. The obtained maximum-likelihood phylogenetic tree of the identified three sequences was clustered with each and with their relevant sequences, as illustrated in Figure 4.

### 3.5. Antimicrobial Susceptibility Profile

The antibiogram findings are outlined in Table 3. *V. alginolyticus* strain showed marked resistance to all antimicrobials used. Furthermore, *A. viridans* strain displayed high resistance to most antibiotics except intermediate sensitivity to amoxycillin/clavulanic acid and ciprofloxacin. However, some isolates were recorded susceptible to antibiotics; for instance, *A. veronii* strain was highly sensitive to amoxycillin/clavulanic acid, oxytetracycline, kanamycin, and trimethoprim/sulfamethoxazole and was intermediately susceptible to ciprofloxacin. In addition, *E. faecalis* strain was also found sensitive to amoxycillin/clavulanic acid, ciprofloxacin, and trimethoprim/sulfamethoxazole and was moderately susceptible to ampicillin and kanamycin. High antibiotic sensitivity was expressed by *Staph. epidermidis* strain to ampicillin, amoxycillin/clavulanic acid, oxytetracycline, novobiocin, and trimethoprim/sulfamethoxazole and it showed intermediate susceptibility to kanamycin and ciprofloxacin.

## 4. Discussion

Tilapia mass mortalities, particularly during summer seasons, represent a puzzling dilemma in Egyptian aquaculture. In this study, deteriorating water quality associated with high water temperatures was observed during the eruption of clinical infections. In addition, fish were cultivated in earthen ponds that were supplied with agriculture drainage water. A total of 30 bacterial isolates were retrieved from cultured *O. niloticus* encountered in heavy mortalities between August 2023 and 2024 in Kafr El-Sheikh province. The bacterial species were recognized as *A. veronii* (30%), *V. alginolyticus* (13.3%), *E. faecalis* (23.3%), *A. viridans* (16.7%)*,* and *Staph. epidermidis* (16.7%). Accordingly, the Gram-positive bacteria were the prevalent group with approximately 57% of the isolates, while the remaining 43% represented the Gram-negative. Prior to 2009, Gram-negative bacteria were the predominant etiological agents of bacterial infections in Egyptian aquaculture. From 2009 to date, Gram-positive bacteria have been frequently isolated in many disease outbreaks [34]. *Str. agalactiae*, *E. faecalis,* and *Lactococcus garvieae* have been reported as the most significant bacterial species causing disease outbreak in Kafr El-Sheikh’s tilapia farms [35]. *E. faecalis* and *A. viridans* were serious causative agents that cause disease problems in tilapia farms in El Fayoum and El Sharkia governorates [36]. This might be attributed to various challenges such as sewage pollution, high organic loads in earthen ponds, and use of untreated poultry manure as pond fertilizers, which could add new pathogens to tilapia production systems [37,38,39].

In the current study, we report four different cases of sudden outbreaks among farmed Nile tilapia along these lines. In the first case, *A. veronii* was recorded in mortality episodes of farmed tilapia in August of 2023. Elgendy et al. [40] reported that *A. veronii* was a critical pathogen causing hemorrhagic septicemia and tilapia mortalities during the summer season in Kafr El-Sheikh. Raised water temperatures, high unionized ammonia levels, and high water pH were considered the most prominent contributing factors that could increase the risk of *A*. *veronii* infection [41,42]. The interaction between this pathogenic bacteria, impaired fish immunity, and poor water quality on initiating summer mortality has been investigated [43].

In the second case, *A. viridans* was recovered from moribund tilapia during mass kills in late spring of 2024. This bacterial infection was firstly reported by Ke et al. [44] in a commercial fishery of tilapia during spring and summer seasons in China. It has emerged as a serious fish pathogen and is considered a new candidate for tilapia aquaculture in Egypt [45]. Elgohary et al. [36] revealed the highest rate of *A. viridans* infection among *O. niloticus* in spring at El Fayoum; however, it had a high prevalence in the summer at El Sharkia governorate.

The mixed infection of *Staph. epidermidis* and *E. faecalis* was detected in several specimens gathered from tilapia mortality in June of 2024. Saleh et al. [34] clarified that *Staph. epidermidis* has been reported as a prevalent pathogen, causing mass mortality in farmed tilapia in Kafr Elsheikh. Those authors hypothesized that the practice of irrigating farms with the same canal water used for wastewater drainage facilitated the spread of *Staph. epidermidis* and reinfection in the affected farms. Another study pointed to their involvement in summer mortalities of tilapia fries within the same geographical area [46]. Furthermore, *E. faecalis* is among the most severe and frequent infections targeting tilapia fish farms, particularly during summer months in Egypt [47,48]. Abdelsalam et al. [19] also documented a case report of mortalities in cultured tilapia and African catfish due to co-infection of *E. faecalis* with *Aeromonas* spp., and *V. alginolyticus*. It was noted that *E. faecalis* could induce severe economic losses in tilapia farms, especially when accompanied by other co-infecting agents. This could be linked to synergetic interactions between two or more bacterial pathogens, which lead to worsening the fish’s health status and accelerating the progression and increasing the intensity of disease [49].

*V. alginolyticus* has been isolated from the mortality outbreak affecting tilapia fish in August of 2024. Coinciding with these results, Ali et al. [50] identified *V. alginolyticus* among bacterial isolates causing summer mortality syndrome of Nile tilapia at different localities, including Kafr El-Sheikh. Previous studies investigated *V. alginolyticus* in cultured *O. niloticus* and Tilapia zillii as well as *V. alginolyticus* and *V. vulnificus* infection in farmed tilapia [51,52]. The epidemiology, geographical spread, and frequency of vibriosis is obviously increased by climate change combined with environmental pollution. This explains why *Vibrio* spp. could thrive and cause disease even in low-salinity environments [53].

In this study, we suppose that higher water temperatures during summer play a key role in these outbreaks. The deleterious effects of elevated water temperature could induce a stressful condition, which successively decreases the fish immunity and increases their susceptibility for infection [54,55]. On top of that, it also allows bacterial pathogens to locate, multiply, and attack stressed fish through different mechanisms [56]. The virulence factors such as adhesions, motility, endotoxins, iron acquisition, proteolytic enzymes, and hemolysins were responsible for the infection scenario of Gram-negative bacteria [57]. On the other hand, the biofilm formation of *Staph. epidermidis* and lipoteichoic acid of *E. faecalis* facilitate their pathogenesis in the affected host [58,59]. Therefore, the virulence factors could enable these pathogens to initiate clinical disease. The affected fish showed similar signs of septicemia, including anorexia, detached scales, skin pigmentation, ulcers, exophthalmia, corneal opacity, fin rot, ascites, and hemorrhages on external body surfaces [60]. The macroscopic findings were similar to those that were listed by Ghetas et al. [61], such as congestion of liver, spleen, and kidney and the abdominal cavity being filled with watery and bloody fluids. Moreover, we tested the hemolytic activity of the isolated bacteria on sheep blood agar and found that *A. veronii* produced α-hemolytic colonies as described by Sadique et al. [62], while this result differs from Sun et al. [63], who found that this strain was β-hemolytic. *V. alginolyticus* showed no hemolysis, as reported in similar previous studies [64,65]. Both *A. viridans* and *E. faecalis* demonstrated α-hemolytic activity [35,36]. In this study, *Staph. epidermidis* showed β-hemolysis [66]; however, it also produced either α-hemolytic colonies [67] or showed no hemolysis [34].

The 16S rRNA sequencing has become a reliable molecular tool for accurate identification and fast diagnosis of bacterial infections [68]. Bacterial isolates were genotypically categorized using sequencing and BLAST analysis of 16S rRNA gene. Sequencing verified that retrieved isolates belonged to the genus *Aeromonas*, *Vibrio*, *Aerococcus*, *Enterococcus*, and *Staphylococcus* spp. By comparing obtained nucleotide sequences, isolates were similar to their related bacteria (>99.5%) and identified as *A. veronii* (PV018985), *V. alginolyticus* (PV016854), *E. faecalis* (PV013413), *A. viridans* (PV032005), and *Staph. epidermidis* (PV012491).

The economic losses associated with summer mortality syndrome are very costly in *O. niloticus* farming in Egypt. Therefore, investigating the etiologies and triggers behind such mortalities is highly required to recommend the best corrective action. Thermal stress accompanied by heatwaves was the major trigger resulting in consequent stress responses, involving decreased D.O. level, elevated toxic ammonia concentrations, oxidative stress, and increasing plasma cortisol. Fish turned immune-compromised and subsequently highly vulnerable to infectious diseases [69]. Tilapia farms relied mainly on agriculture drainage water and treated wastewater that contain various bacterial pathogens, specifically aeromonads, *Vibrios*, and enterococci, causing health hazards to fish and aquatic animals [38,70]. As a result, these bacteria are recognized as opportunists that can invade stressed fish and initiate the disease [71]. Ali et al. [12] declared that there was a significant correlation between water sources (surface and irrigation canals) and both the incidence and level of unusual tilapia mortalities. Intensification also contributed to tilapia disease outbreaks worldwide, with bacterial infections resulting in morbidities, mortalities, and influencing sustainable production [72]. So, bad farming practices and biosecurity measures have a crucial role in disease occurrence [73].

Antibiotics are used violently in agricultural activities, including in aquaculture, humans, and livestock, making them ubiquitous in aquatic ecosystems, posing serious health risks to the exposed aquatic animals and humans [74,75]. The antibiotic sensitivity (antibiogram) test is usually carried out to evaluate the susceptibility of bacteria to field-used antibiotics. The bacterial isolates retrieved from diseased Nile tilapia farmed in Kafr El-Sheikh province, Egypt, showed variability to the antibiogram tests. For instance, *A. hydrophila*, *Ps. fluorescens*, and *Str. iniae* isolated from diseased Nile tilapia showed high sensitivity to ciprofloxacin [20]. According to El-Gohary et al. [76], most of the isolated aeromonad species displayed the highest resistance to chloramphenicol, azithromycin, and kanamycin, while showing lower resistance against streptomycin, amoxicillin, and cefotaxime. These differences may be attributed to the presence of different antibiotic resistance genes in the bacterial isolates.

Our study emphasized that most bacterial strains revealed a notable resistance to tested antibiotics. *V. alginolyticus* strain showed high resistance against all antibacterials: penicillin, ampicillin, ciprofloxacin, oxytetracycline, erythromycin, novobiocin, amoxycillin/clavulanic acid, trimethoprim/sulfamethoxazole, and kanamycin. Moreover, *A. viridans* strain was resistant to all antibiotics except for intermediate sensitivity to amoxycillin/clavulanic acid and ciprofloxacin. According to previous review, multiple registered antibiotics were available to combat bacterial infections in Mediterranean finfish farming, including fluoroquinolones, tetracyclines, penicillin, potentiated sulfa, and chloramphenicol. The same authors explained that oxytetracycline and quinolone drugs are the most used antibiotics in aquaculture [77]. Sulfonamides (sulfamethoxazole or the combined form sulfamethoxazole/trimethoprim) represent the third most prevalent antimicrobial after tetracyclines and quinolones used in fish farming [78]. Recently, Elsherbiny et al. [79] confirmed that tetracyclines, quinolones, and sulfonamides are major antimicrobial classes used in aquaculture. The inappropriate use of drugs or inaccurate doses derived from rushed diagnosis without any veterinarian supervision will lead to the presence of bacterial resistance to these antimicrobials [80]. Conversely, *A. veronii* strain showed high sensitivity to trimethoprim/sulfamethoxazole, amoxycillin/clavulanic acid, oxytetracycline, and kanamycin and moderate sensitivity to ciprofloxacin. These findings align with El Latif et al. [81], who presented that *A. veronii* was only sensitive to sulfamethoxazole–trimethoprim and ofloxacin and resistant to ampicillin, gentamycin, lincomycin, tetracycline, and oxytetracycline. Another study established that this strain was sensitive to florfenicol, sulfamethoxazole–trimethoprim, ciprofloxacin, chloramphenicol, enrofloxacin, and nitrofurantoin and resistant to penicillin, ampicillin, and amoxicillin [42]. Our study demonstrated that *E. faecalis* strain was sensitive to trimethoprim/sulfamethoxazole, amoxycillin/clavulanic acid, and ciprofloxacin and was partially susceptible to ampicillin and kanamycin. These results agree with Abu-Elala et al. [35], in which *E. faecalis* was susceptible to sulfamethoxazole/trimethoprim and penicillin, intermediately resistant to amoxicillin/clavulanic acid and ciprofloxacin but resistant to ampicillin, erythromycin, neomycin, streptomycin, and gentamycin. Abdel-moneam et al. [82] declared that this isolate was sensitive to ciprofloxacin, trimethoprim–sulfamethoxazole, levofloxacin, chloramphenicol, and penicillin. In addition, *Staph. epidermidis* strain exhibited a high degree of sensitivity to ampicillin, amoxycillin/clavulanic acid, oxytetracycline, novobiocin, and trimethoprim/sulfamethoxazole and intermediate susceptibility to kanamycin and ciprofloxacin. These results seem to be consistent with Kubilay and Uluköy [66], who found that this strain was sensitive to ciprofloxacin, trimethoprim, sulfamethoxazole, norfloxacin, and chloramphenicol and resistant to erythromycin, penicillin, ampicillin–sulbactam, ampicillin, gentamycin, oxytetracycline, and streptomycin. Finally, these antibiotics that showed sensitivity could be recommended for control during the peak of disease only. However, vaccination programs, probiotics, and enhanced production systems are three examples that are considered valid alternative practices to reduce the use of antibiotics in aquaculture [78].

## 5. Conclusions

The present study reported five bacterial strains, *A. veronii*, *V. alginolyticus*, *E. faecalis*, and *Staph. Epidermidis*, that have been isolated and characterized from different mass tilapia mortalities during summer months of 2023 and 2024 in Kafr El-Sheikh province, Egypt. High water temperatures during summer may contribute to increasing the risk of these infections. *E. faecalis* and *Staph. epidermidis* have been occurring as bacterial co-infections and appear to result in high mortality rates and significant economic losses in farmed fish. Moreover, *A. viridans* has been identified as an emerging bacterial pathogen for tilapia aquaculture. The most worrying aspect is the high antibiotic resistance in the isolated strains against most of the tested antibiotics. Thus, it is crucial to perform antibiogram testing prior to administering antibiotics in aquaculture to guarantee effective treatment and to minimize the emergence of antibiotic-resistant strains.

## Figures and Tables

**Table 1:** Outbreaks and the sources of bacterial isolates retrieved from diseased Nile tilapia (*Oreochromis niloticus*).

Cases	Date of Collection	No. of Examined Samples	No. of Isolates	% *	Tissue Samples		
					Liver	Kidney	Spleen
Outbreak No. 1	August 2023	10	9	30.0	4	2	3
Outbreak No. 2	April 2024	10	5	16.7	2	2	1
Outbreak No. 3	June 2024	12	12	40.0	5	3	4
Outbreak No. 4	August 2024	8	4	13.3	2	1	1
Total		40	30	100	13	8	9

* The percentage of isolation in relation to the total number of bacterial isolates.

**Table 2:** Phenotypic and biochemical characteristics of the recovered bacterial isolates.

Tests	*A. veronii*	*V. alginolyticus*	*A. viridans*	*E. faecalis*	*Staph. epidermidis*
Colony on NA or BHIA	Smooth, round, convex creamy white colonies on NA	Swarmed white colonies on NA	Pinpoint white colonies on BHIA	Small, smooth, flat, grayish-white colonies on BHIA	Round, opaque, white colonies on BHIA
Gram staining	Gram-negative	Gram-negative	Gram-positive	Gram-positive	Gram-positive
Shape	Short bacilli	Curved rods	Cocci arranged in long chain	Cocci arranged in pairs or clusters	Cocci formed grape-like clusters
Colony on TCBS	Small round, light yellow	Large convex, yellow-colored	ND	ND	ND
Hemolysis on blood agar	α	γ	α	α	β
Motility	+	+	-	-	-
Cytochrome oxidase	+	+	-	-	-
Catalase	+	+	-	-	+
TSI test	K/A (no gas)	A/A (no gas)	ND	ND	ND
Growth in 4% NaCl	+	+	+	+	+
Growth in 6% NaCl	+	+	+	+	+
Growth in 8% NaCl	+	+	+	+	+
Growth in 10% NaCl	-	-	-	-	-

(+): positive; (-): negative; (A/A): acidic/acidic; (K/A): alkaline/acidic; (ND): not done; (α): Alpha hemolysis; (β): Beta hemolysis; (γ): Gamma hemolysis.

**Table 3:** Results of the antibiotic sensitivity test using the disc diffusion method.

Antibiotic	Code (Number Indicates Concentration in μg Per Disc)	Bacterial Isolates				
		*A. veronii*	*V. alginolyticus*	*A. viridans*	*E. faecalis*	*Staph. epidermidis*
Penicillin	P^10^	R	R	R	R	R
Ampicillin	AMP^10^	R	R	R	I	S
Amoxycillin/Clavulanic acid	AMC^20/10^	S	R	I	S	S
Trimethoprim/Sulfamethoxazole	COT^25^	S	R	R	S	S
Ciprofloxacin	CIP^5^	I	R	I	S	I
Oxytetracycline	O^30^	S	R	R	R	S
Erythromycin	E^15^	R	R	R	R	S
Novobiocin	NV^30^	R	R	R	R	S
Kanamycin	K^30^	S	R	R	I	I

(R): Resistant; (I): Intermediate; (S): Sensitive.

## Data Availability

The original contributions presented in this study are included in the article/Supplementary Material. Further inquiries can be directed to the corresponding author.

## Supplementary Materials

The following supporting information can be downloaded at: https://www.mdpi.com/article/10.3390/microorganisms13112448/s1, Figure S1. PCR amplification of the 16S rRNA gene of Gram-negative bacterial strains isolated from diseased Nile tilapia (*Oreochromis niloticus*). Lane (**P**): the control positive sample; lane (**N**): the negative control sample; 100 bp DNA ladder; and lane (**V1**): the *Vibrio* sample. The PCR products shown correspond to the predicted molecular mass of 663 bp (16S rRNA gene). In addition, lane (**A**) represents the *Aeromonas* sample. The PCR products shown correspond to the predicted molecular mass of 953 bp (16S rRNA gene). Figure S2. PCR amplification of the universal 16S rRNA gene of Gram-positive bacterial strains isolated from diseased Nile tilapia (*Oreochromis niloticus*). Lane (**P**): the control positive sample; lane (**N**): the negative control sample, 100 bp DNA ladder; lane (**1**): the *Enterococcus faecalis* sample; lane (**2**): the *Staphylococcus epidermidis* sample; and lane (**8**): the *Aerococcus viridans* sample. The PCR products shown correspond to the predicted molecular mass of 1485 bp (16S rRNA gene). Table S1: Primers used in this study. Table S2. Biochemical profile of bacterial isolates using VITEK 2 with 97% probability of *E. faecalis* and 99% probability of *Staph. epidermidis.*
