# Supplementary material for: Bacterial Pathogens and Their Antimicrobial Resistance in Farmed Nile Tilapia Experiencing “Summer Mortality” in Kafr El-Sheikh, Egypt
Source: Microorganisms. 2025 Oct 25;13(11):2448. doi: 10.3390/microorganisms13112448 (PMC12654396; doi:10.3390/microorganisms13112448)
Supplement: Supplementary file 1 [file microorganisms-13-02448-s001.zip › microorganisms-3914226-supplementary.pdf]

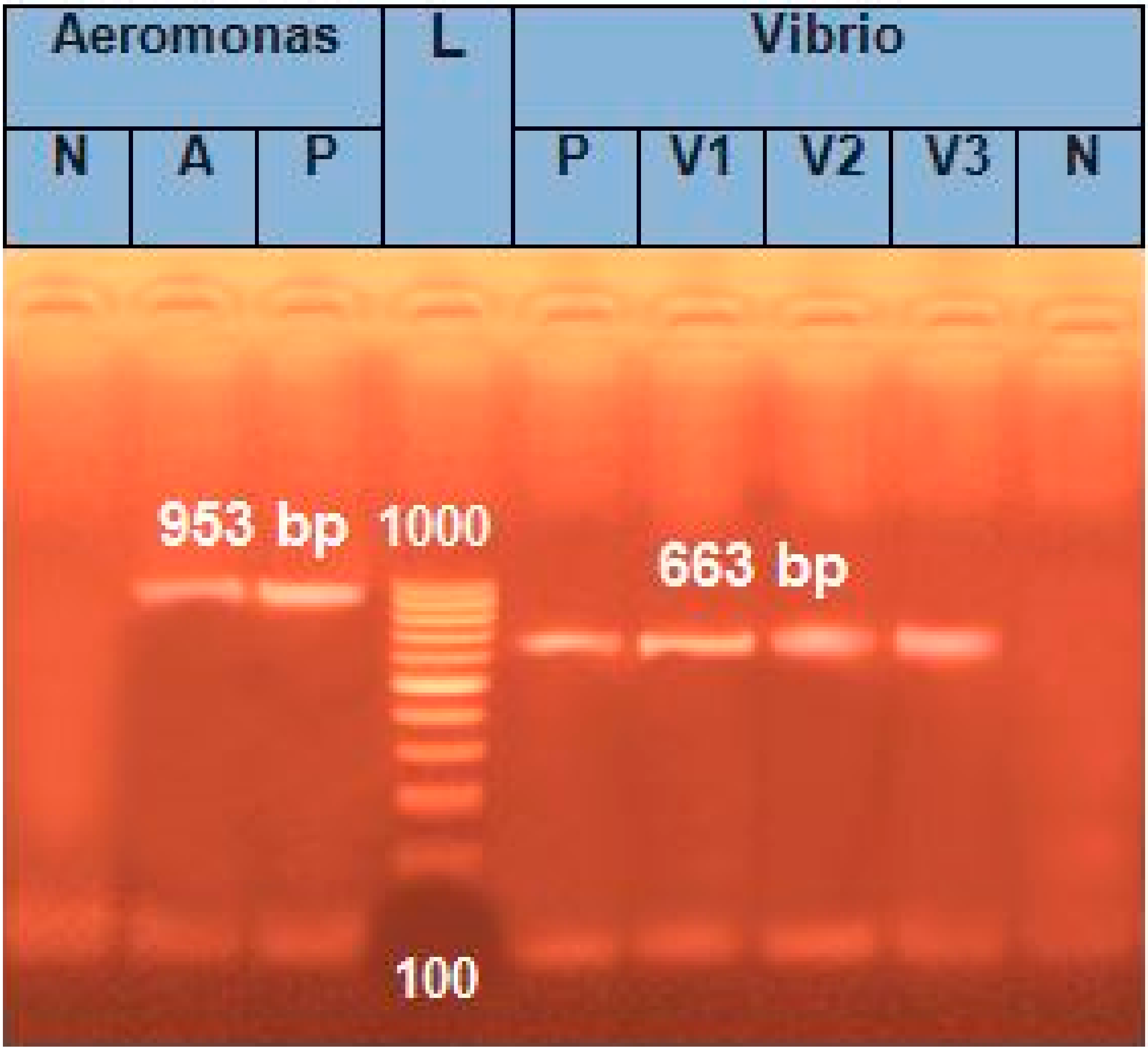

Figure S1

PCR amplification of the 16S rRNA gene of Gram-negative bacterial strains isolated from diseased Nile tilapia (*Oreochromis niloticus*). Lane (**P**): the control positive sample; lane (**N**): the negative control sample; 100 bp DNA ladder; and lane (**V1**): the *Vibrio* sample. The PCR products shown correspond to the predicted molecular mass of 663 bp (16S rRNA gene). In addition, lane (**A**) represents the *Aeromonas* sample. The PCR products shown correspond to the predicted molecular mass of 953 bp (16S rRNA gene).

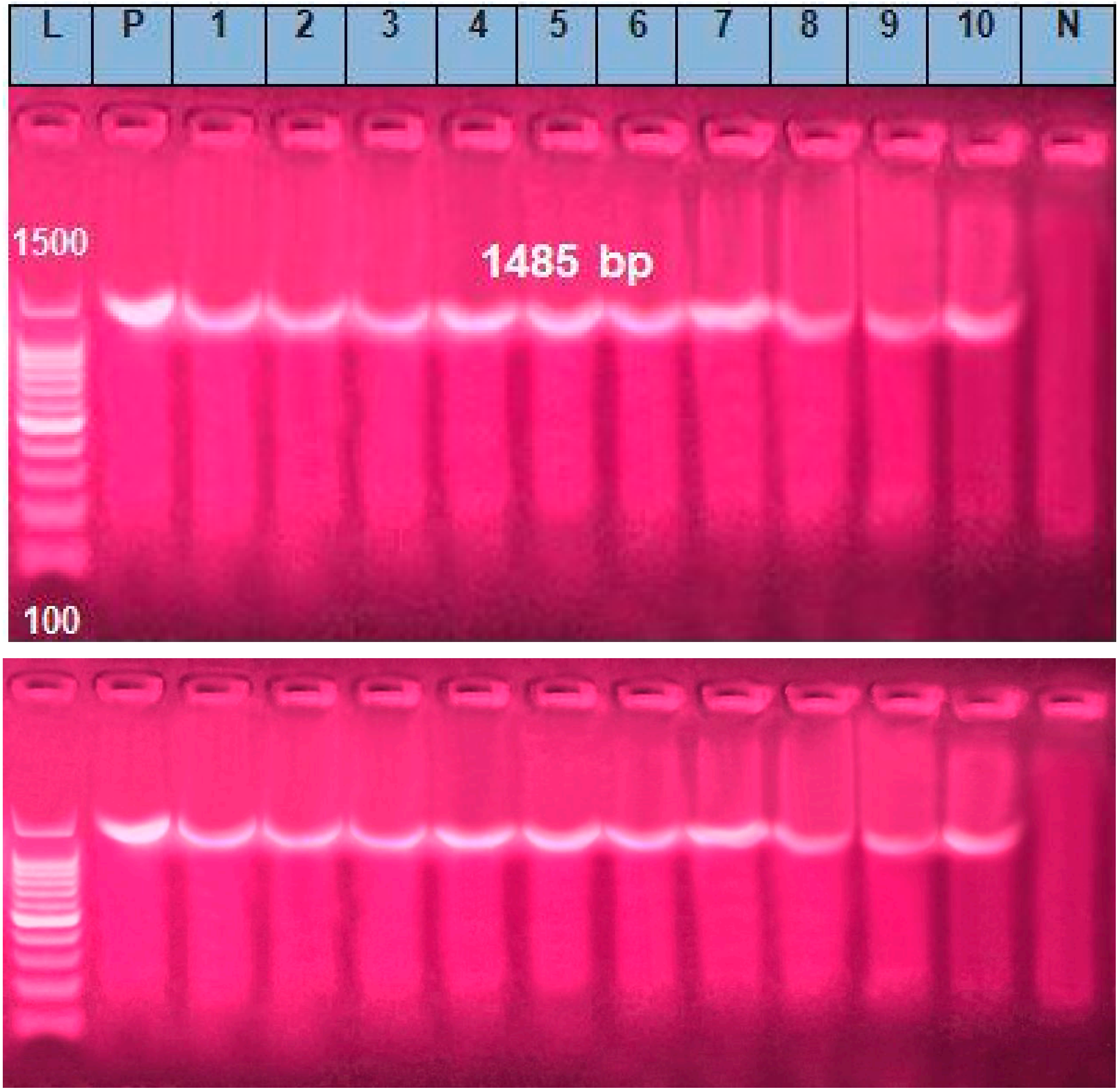

Figure S2

PCR amplification of the universal 16S rRNA gene of Gram-positive bacterial strains isolated from diseased Nile tilapia (*Oreochromis niloticus*). Lane (**P**): the control positive sample; lane (**N**): the negative control sample, 100 bp DNA ladder; lane (**1**): the *Enterococcus faecalis* sample; lane (**2**): the *Staphylococcus epidermidis* sample; and lane (**8**): the *Aerococcus viridans* sample. The PCR products shown correspond to the predicted molecular mass of 1485 bp (16S rRNA gene).

Table S1 (Supplementary Material).

Primers used in PCR in this study.

Primer	Primer sequences	Specificity	Size of amplified product (bp)	References
Universal 16S primer	AGAGTTTGATCMTGGCTCAG<br>TACGGYTACCTTGTTACGACTT	Bacterial pathogens	1485	(Lagacé et al., 2004)
Aeromonas 16S rRNA	CTACTTTTGCCGGCGAGCGG<br>TGATTCCCGAAGGCACTCCC	*Aeromonas* spp.	953	(Gordon et al., 2007)
Vibrio 16S rRNA	CGGTGAAATGCGTAGAGAT<br>TTACTAGCGATTCCGAGTTC	*Vibrio* spp.	663	(Tarr et al., 2007)

Table S2 (Supplementary Material).

Biochemical profile of bacterial isolates using VITEK 2 with 97% probability of *E. faecalis* and 99% probability of *Staph. epidermidis*.

	Biochemical test	Abbreviation	*E. faecalis*	*Staph. epidermidis*
2	D-amygdalin	AMY	+	-
4	Phosphatidylinositol phospholipase C	PIPLC	-	-
5	D-xylose	dXYL	-	-
8	Arginine dihydrolase 1	ADH1	+	+
9	Beta-galactosidase	BGAL	-	(+)
11	Alpha-glucosidase	AGLU	-	-
13	Ala-Phe-Pro arylamidase	APPA	-	-
14	Cyclodextrin	CDEX	+	-
15	L-aspartate arylamidase	AspA	+	-
16	Beta galactopyranosidase	BGAR	-	-
17	Alha-mannosidase	AMAN	-	-
19	Phosphatase	PHOS	-	+
20	Leucine arylamidase	LeuA	-	-
23	L-proline arylamidase	ProA	-	-
24	Beta glucuronidase	BGURr	-	-
25	Alpha-galactosidase	AGAL	-	-
26	L-pyrrolidonyl-arylamidase	PyrA	+	-
27	Beta-glucuronidase	BGUR	-	-
28	Alanine arylamidase	AlaA	+	-
29	Tyrosine arylamidase	TyrA	+	-
30	D-sorbitol	dSOR	+	-
31	Urease	URE	-	+
32	Polymixin b resistance	POLYB	+	-
37	D-galactose	dGAL	+	+
38	D-ribose	dRIB	+	-
39	L-lactate alkalinization	ILATk	-	+
42	Lactose	LAC	-	+

44	N-acetyl-d-glucosamine	NAG	+	-
45	D-maltose	dMAL	+	+
46	Bacitracin resistance	BACI	+	+
47	Novobiocin resistance	NOVO	+	-
50	Growth in 6.5% NaCl	NC 6.5	+	+
52	D-mannitol	dMAN	+	-
53	D-mannose	dMNE	+	+
54	Methyl-B-D-glucopyranoside	MBdG	+	-
56	Pullulan	PUL	-	-
57	D-raffinose	dRAF	-	-
58	O/129 resistance	O129R	+	+
59	Salicin	SAL	+	-
60	Saccharose/sucrose	SAC	+	+
62	D-trehalose	dTRE	+	-
63	Arginine dihydrolase 2	ADH2s	+	-
64	Optochin resistance	OPTO	+	+
